# Epidemiology and time trends of distal forearm fractures in adults - a study of 11.2 million person-years in Sweden

**DOI:** 10.1186/s12891-017-1596-z

**Published:** 2017-06-02

**Authors:** Daniel Jerrhag, Martin Englund, Magnus K. Karlsson, Bjorn E. Rosengren

**Affiliations:** 1 Clinical and Molecular Osteoporosis Research Unit, Departments of Clinical Sciences and Orthopedics Malmö, Skåne University Hospital, Lund University, SE 20502 Malmo, Sweden; 2 Department of Clinical Sciences Lund, Orthopedics, Clinical Epidemiology Unit, Lund University, Faculty of Medicine, Lund, Sweden; 3 Clinical Epidemiology Research and Training Unit, Boston University School of Medicine, Boston, MA USA

**Keywords:** Epidemiology, Time-trends, Wrist fracture, Distal radius fracture, Forecast

## Abstract

**Background:**

A distal forearm fracture is a very common injury causing both suffering and substantial health care costs. The incidence of this fracture type seemed to increase worldwide until the middle 1980’s, but thereafter most reports have shown stable or decreasing rates. As few large studies have been presented lately we aimed to describe recent epidemiology and time trends of distal forearm fractures in adults. We paid special attention to fractures in working ages as they present challenges in terms of treatment and costs for sick-leave, and have not previously been thoroughly investigated.

**Methods:**

By use of population data from Statistics Sweden and official in- and out-patient register data of men and women (≥17 years) in Sweden (Skåne region), we ascertained distal forearm fractures and estimated age- and sex-specific rates and time-trends from year 1999 to 2010 (11.2 million person-years (py)).

**Results:**

The total incidence rate was 278 per 100,000 py (31,233 fractures) with 23% higher annual numbers 2010 compared with 1999. An increase in the annual age standardized incidence was found in men, +0.7% per annum (95% confidence interval (CI) 0.1, 1.4), and women, +0.9% (95% CI 0.5, 1.3), driven mainly by an increasing incidence in working ages (17–64 years). Also, expected demographic changes including a 25% population increase may result in 38% more fractures until 2050, compared to 2017.

**Conclusions:**

The incidence of distal forearm fractures in adults in southern Sweden is increasing, mainly driven by an increase in working ages. In combination with expected demographic changes these findings may present substantial challenges for the future.

## Background

The distal forearm fracture is a common orthopedic injury. The consequences for the individual patient depend on factors such as age, fracture pattern and occupation. In epidemiological studies the age rate curve has been found to be bimodal, with highest incidences found in children and elderly [1, 2].

In young adulthood and middle age the distal forearm fracture is less common than in childhood and in the elderly, but fracture patterns can be severe with consequent loss of function or absence from work. The higher incidence rates of this fracture type in the elderly result in substantial suffering as well as health care costs.

We have earlier found that the incidence of distal forearm fractures among children is increasing in the Skåne region, Sweden [3]. In adults, however, few larger studies of distal forearm fracture epidemiology have been presented recently [4, 5] and none with focus on working-age individuals. To provide politicians and public health planners with current data, we aimed to, in the Skåne region, Sweden (i) describe present epidemiology of distal forearm fractures in the adult population, specifically also in the working-age population, (ii) estimate time-trends during the most recent decade, and (iii) make a forecast to estimate the number of fractures the decades to come.

## Methods

The *Skåne Healthcare Register (SHR)* is a register covering in- and outpatient health care provided to residents of Skåne, the southernmost region of Sweden from year 1998. The region includes both urban and rural areas and had in year 2010 a total adult (≥17 years) population of about 1.0 million. From year 1999 to 2010 we used the SHR to ascertain distal forearm fractures in adults (≥17 years) residing in the region (11.2 million py) by using physician-set diagnostic codes according to the Swedish version of the International Classification of Diseases (ICD) 10 system (S52.50, S52.51, S52.60, S52.61). Bilateral fractures were counted as only one fracture as the database does not include information about side. The washout period was set to 1 year (365 days) for each fracture and unique individual and we consequently also included year 1998 data to create a reference for washouts year 1999. From the complete data set we estimated sex-specific incidence rates per 100,000 py using the cumulative annual adult population, in one year age classes from Statistics Sweden as denominator (population at risk) [6]. For estimation of temporal trends, we tabulated data by year and used Poisson regression of annual direct age-standardized incidence rates (with the average population during the examined years as the standard population) and included 95% confidence intervals (95% CI) to describe uncertainty. The validity of the register, in terms of distal forearm fractures, has previously been examined with a sensitivity of 90% and positive predictive value of 94% for the register data compared to gold standard [7].

To estimate the number of distal forearm fractures in the whole of Sweden the forthcoming decades, the overall incidence in 1-year age groups during the examination period from 1999 to 2010 from the Skåne region was applied to demographic forecasts from Statistics Sweden for the years 2017–2050 [8].

We used SAS system v 9.2, SPSS v17.0, and Microsoft Excel 2003 for data management and statistical calculations. All tests were two-tailed and we considered a *p*-value lower than 0.05 to be statistically significant. The study has been approved by the Ethical Review Board (ERB) at Lund University (2011/432).

## Results

From year 1999 to 2010 we identified 31,233 distal forearm fractures (male to female ratio 1:3) during 11.2 million py. This represents an overall incidence of 278 per 100,000 py (Table 1).

**Table 1:** Population at risk, number of fractures and overall wrist fracture incidence

Age stratum (years)	Men			Women		
	At risk	Fractures	Incidence	At risk	Fractures	Incidence
≥ 17	5,478,970	8217	150	5,762,155	23,016	399
17–64	4,412,748	6045	137	4,342,588	9007	207
≥ 65	1,066,222	2172	204	1,419,567	14,009	987
17–49	3,081,881	4106	133	3,016,896	3425	114
≥ 50	2,397,089	4111	171	2,745,259	19,591	714
17–19	264,337	781	295	255,140	343	134
20–29	897,345	1152	128	889,840	835	94
30–39	983,138	946	96	951,017	954	100
40–49	937,061	1227	131	915,592	1293	141
50–59	920,560	1287	140	916,685	3201	349
60–69	734,078	1117	152	757,112	4699	621
70–79	476,240	843	177	588,380	5269	896
80–89	235,815	717	304	399,501	4989	1249
≥90	30,398	147	484	88,891	1433	1612

Different age-strata (years) and incidence per 100,000 py during 1999–2010 in the Skåne region, Sweden

Adults in working ages (17–64 years) contributed to 15,052 fractures (48% of all fractures, male to female ratio 1:1.5). Correspondingly, those older than 64 years contributed to 16,181 fractures (52% of all, male to female ratio 1:6.5). From 1999 to 2010 the overall annual number of distal forearm fractures increased from 2368 to 3089, with underlying increases in both men (from 589 to 766) and women (from 1779 to 2323). An additional file shows the number of persons at risk, and the number of fractures more in detail (additional file 1).

There was also a significant increase in the annual age-standardized incidence in both men (+0.7% per annum, 95% CI 0.1, 1.4), and women (+0.9%, 95% CI 0.5, 1.3). For adults in working ages (17–64 years) the incidence increased significantly for both men (+0.8%, 95% CI 0.02, 1.5) and women (+2.1%, 95% CI 1.5, 2.8) while it was stable in their counterparts ≥65 years. Detailed data including other relevant age groups are presented in Table 2.

**Table 2:** Annual change in age standardized rates in different age-strata in the Skåne County 1999–2010

	Annual percent change (95% CI)	
Age stratum (years)	Men	Women
Primary analyses		
≥ 17	**+0.7 (0.1, 1.4)**	**+0.9 (0.5, 1.3)**
17–64	**+0.8 (+0.0, 1.5)**	**+2.1 (1.5, 2.8)**
≥ 65	+0.5 (−0.7, 1.8)	+0.1 (−0.3, 0.6)
Secondary analyses		
17–49	+0.7 (−0.2, 1.6)	+1.0 (−0.0, 1.9)
≥ 50	+0.8 (−0.1, 1.7)	**+0.5 (0.1, 0.9)**
Tertiary analyses		
17–19	+1.7 (−0.4, 3.8)	+2.3 (−0.8, 5.5)
20–29	+1.3 (−0.4, 3.0)	+0.0 (−1.9, 2.0)
30–39	+0.4 (−1.4, 2.3)	−0.5 (−2.3, 1.3)
40–49	−0.2 (−1.8, 1.4)	**+2.3 (0.7, 4.0)**
50–59	**+2.0 (0.4, 3.7)**	**+3.4 (2.3, 4.4)**
60–69	−0.7 (−2.4, 1.0)	**+1.7 (0.9, 2.6)**
70–79	+0.0 (−1.9, 2.0)	**+2.0 (1.2, 2.9)**
80–89	+2.1 (−0.1, 4.3)	+0.2 (−0.6, 1.0)
≥ 90	−0.8 (−5.4, 4.0)	+0.6 (−0.9, 2.1)

For clarity rather than for precision one decimal is given. Statistically significant changes are bolded

**Fig. 1:**
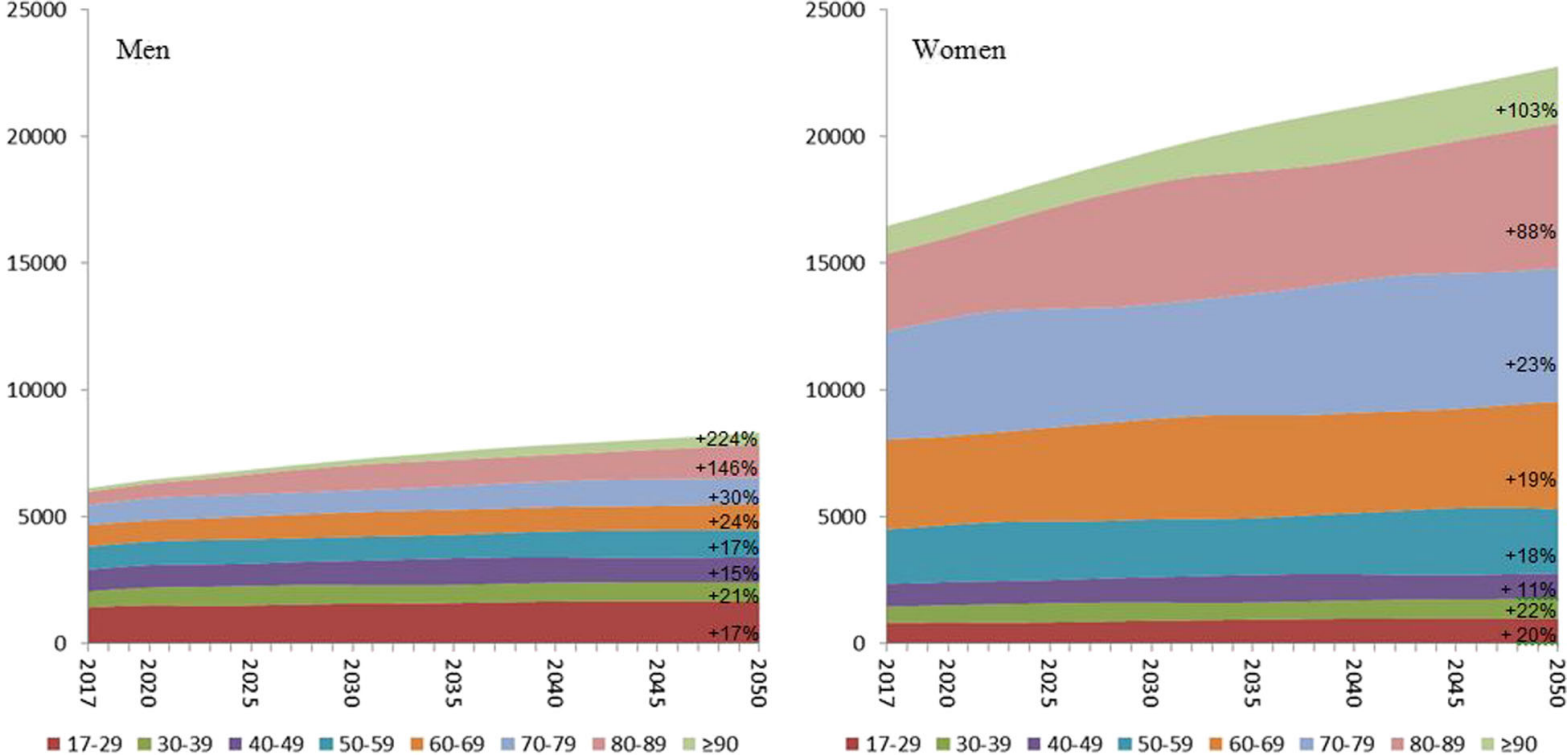
Projection of numbers of distal forearm fractures in Sweden the forthcoming decades (2017–2050). Age strata in years. Change in % from 2017 to 2050. Over all incidence in 1-year age groups in Skåne region, Sweden 1999–2010 used for calculation (see methods)

**Fig. 2:**
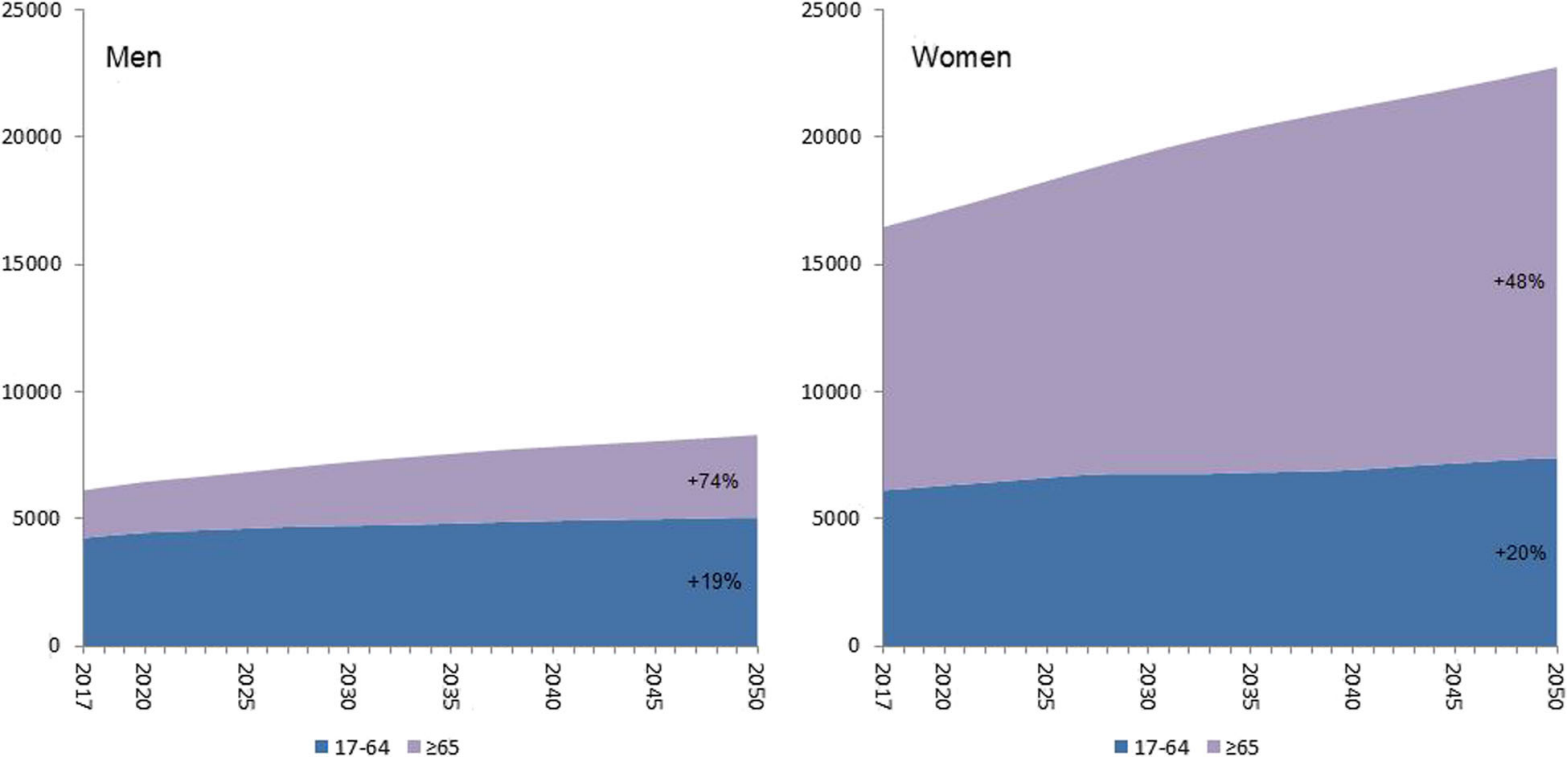
Projection of numbers of distal forearm fractures in Sweden in working and non-working ages. Age strata in years. Change in % from 2017 to 2050. Over all incidence in 1-year age groups in Skåne region, Sweden 1999–2010 used for calculation (see methods)

Even though the Swedish population ≥ 17 years is estimated to increase by only 25% from 8,110,000 (year 2017) to 10,100,000 (year 2050) we project an increase in number of forearm fractures by 38% from 22,600 (year 2017) to 31,000 (year 2050) due to shifts in age structure (Fig. 1). In working ages (17–64 years) the total number of fractures is projected to increase from 10,400 to 12,500, representing an increase by 20%. In the age-group ≥65 years the number of fractures is projected to increase from 12,200 to 18,600, an increase by 52% (Fig. 2).

## Discussion

In our study of 11.2 million py in a well-defined Swedish population we found that the age-standardized incidence in both adult (≥17 years) men and women increased significantly from year 1999 to 2010. This was driven mainly by an increase in working ages (17–64 years). With an increasing population and the expected demographic changes until year 2050 we project the number of distal forearm fractures to increase by 38% in Sweden from 2017 to year 2050, even though the actual number of citizens is only estimated to increase by 25%.

**Fig. 3:**
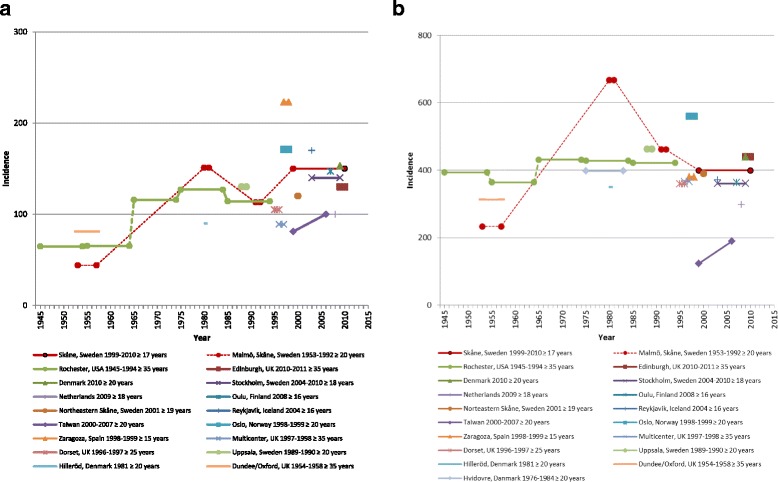
**a**. Reported incidence of distal forearm fractures in men per 100,000 py during the last 6 decades. **b**. Reported incidence of distal forearm fractures in women per 100,000 py during the last 6 decades. [2, 4, 5, 9, 11, 13–18, 21, 22, 39–45]

Distal forearm fracture rates have been superintended episodically and most studies agree on an increase from the 1950’s to the 1980’s [2, 9–12]. After the 1980’s some studies have reported decreasing or stable adult distal forearm fracture rates [5, 13–20], whilst some recent reports, from Denmark and Taiwan, infer an increasing incidence [4, 21] (Fig. 3a, b).

A new large British study [20] during years 1988–2012 however reports a lower incidence in the entity radius/ulnar fractures (not only distal forearm fractures) than that we found for only distal forearm fractures. Since definition of fracture, ascertainment method, geographical regions and age stratification differ substantially between studies, direct comparisons are difficult (Tables 3 and 4).

**Table 3:** Reported incidence of distal forearm fractures in adults (≥ 50 years) during 7 decades

Population at risk	Year	Incidence	
		Men	Women
Skåne, Sweden^a^	1999–2010	171	712
Denmark^a^ [4]	2010	203	926
Austria^b^ [46]	2010	162	607
Oulu, Finland^a^ [16]	2008	223	710
Netherlands^a^ [44]	2009	147	612
Kristiansand, Norway^b^ [47]	2004–2005	189	751
Northeastern Skåne, Sweden^a^ [22]	2001	152	677
Austria^b^ [46]	1999	171	709
Oslo, Norway^a^ [14]	1998–1999	254	1098
Malmö, Sweden^a^ [13]	1991–1992	157	827
Uppsala, Sweden^a^ [42]	1989–1990	217	970
Malmö, Sweden^ac^ [9]	1980–1981	158	462
Hvidovre, Danmark^a^ [45]	1976–1984	-	695
Hilleröd, Denmark^a^ [11]	1981	116	824
Oslo, Norway^a^ [40]	1979	234	1137
Malmö, Sweden^a^ [2]	1953–1957	53	510

Incidence per 100,000 py

^a^Crude incidence

^b^Age standardized incidence

^c^Numbers derived from Jónsson et al. [13]

**Table 4:** Reported incidence of distal forearm fractures in adults in different countries or areas

Population at risk	Year	Age (Years)	Incidence	
			Men	Women
Skåne, Sweden^a^	1999–2010	≥ 17	150	399
Edinburgh, UK^a^ [41]	2010–2011	≥ 35	130	440
Denmark^a^ [4]	2010	≥ 20	153	530
Stockholm, Sweden^a^ [5]	2004–2010	≥ 18	140	360
Oulu, Finland^a^ [16]	2008	≥ 16	147	363
Netherlands^a^ [44]	2009	≥ 18	100	298
Taiwan^a^ [21]	2007	≥ 20	100	189
Reykjavik, Iceland^a^ [17]	2004	≥ 16	170	370
Northeastern Skåne, Sweden^a^ [22]	2001	≥ 19	120	390
Taiwan^a^ [21]	2000	≥ 20	81	123
Oslo, Norway^a^ [14]	1998–1999	≥ 20	171	560
Dorset, UK^a^ [18]	1996–1997	≥ 25	105	359
Zaragoza, Spain^a^ [39]	1998–1999	≥ 15	223	380
Multicenter, UK^a^ [43]	1997–1998	≥ 35	90	368
Malmö, Sweden^a^ [13]	1991–1992	≥ 20	113	461
Rochester, Minnesota^b^ [15]	1985–1994	≥ 35	114	421
Uppsala, Sweden^a^ [24]	1989–1990	≥ 20	130	463
Rochester, Minnesota^b^ [15]	1975–1984	≥ 35	127	428
Hvidovre, Danmark^a^ [45]	1976–1984	≥ 20	c	397
Malmö, Sweden^ad^ [9]	1980–1981	≥ 20	140	667
Hilleröd, Denmark^a^ [11]	1981	≥ 20	90	350
Oslo, Norway^a^ [40]	1979	≥ 20	169	673
Rochester, Minnesota^b^ [15]	1965–1974	≥ 35	116	432
Rochester, Minnesota^b^ [15]	1955–1964	≥ 35	65	364
Dundee/Oxford, UK^af^ [41]	1954–1958	≥ 35	81	313
Rochester, Minnesota^b^ [15]	1945–1954	≥ 35	65	393
Malmö, Sweden^ad^ [2]	1953–1957	≥ 20	44	233

Incidence per 100,000 py

^a^Crude incidence

^b^Age standardized incidence

^c^No male figures given

^d^Numbers derived from Jónsson et al. [13]

^f^Numbers derived from Court-Brown et al. [41]

We therefore primarily compared our results to earlier reports from the Skåne region, Sweden [2, 9, 13] (Fig. 3a, b). Jonsson et al. [13] reported a lower incidence in both genders in the adult population in Malmö (the largest city in the Skåne region) 1991–1992 compared to 1980–1981. In our data from the period 1999–2010, the incidence in men ≥17 years (150 per 100,000 py) were at the level of those for Malmö 1980–1981 but higher than those from 1991 to 1992. In contrast, for women, the incidence was lower during 1999–2010 (339 per 100,000 py), than in Jonsson et al. 1991–1992 [13]. In the north-eastern part of the Skåne region, Brogren et al. [22] reported an incidence in men ≥19 years of 120 per 100,000 py and in women of 390 per 100,000 py in year 2001. In Finland, Flinkkilä et al. reported an incidence for men and women ≥16 years, of 147 and 363 per 100,000 py, respectively, in year 2008. Both Brogren et al. and Flinkkilä et al. base their results on fracture ascertainment through radiographs and medical charts and their results are similar to ours.

In our study we found a significant annual increase in the age standardized distal forearm fracture rate in adult men and women. As a distal forearm fracture has been found to be an indicator of osteoporosis [23] and also forecast subsequent fractures, [24, 25] these results may have implication for the future fracture burden. Our results are in some contrast to Wilcke et al. [5] who in Stockholm, Sweden, found a decreasing forearm fracture rate from year 2004 to 2010 in individuals ≥65 years of age. However, their findings must be interpreted with care as they included only one fracture per individual, rendering a decreasing population at risk with successive years without a corresponding decrease in the denominator and without adjustment for lower risks in those who remained eligible. On the other hand Abrahamsen et al. [4] in a recent large register based study in Denmark, found a higher incidence compared to earlier. Their reported incidence for men (153 per 100,000 py) was nearly the same as we found, but for women (530 per 100,000 py) somewhat higher (Fig. 3a, b).

The reasons behind the increase in incidence we found during 1999–2010 are not examined in this study but several factors may have contributed including an increased prevalence of osteoporosis. Trend data available for osteoporosis in the Skåne region, Sweden [26–28], do however not indicate this. More recent studies from other countries including Finland and USA, actually point in the opposite direction with an increasing time trend in bone mineral density (BMD) [29, 30]. A more strict postmenopausal estrogen prescription compared to earlier may have contributed to an increased fracture incidence but this effect is likely small as the incidence is increasing in both men and women. In Sweden, as in many other countries, body mass index (BMI) is increasing [31]. The importance of BMI for fracture risk has been known since long but recent insights have inferred that a higher BMI, apart from giving a lower risk for hip fracture, also confers a higher risk for upper extremity fracture [32].

Apart from consequences for the individual there are other implications of fractures. As the distal forearm fracture is the most common of fractures, even a minor increase in occurrence could substantially impact health care resource demands and public health costs. This would be especially true if the increase occurs in working ages where patients need sick-leave, depending on occupation, for 5–12 weeks or more. Any permanent loss of function may also result in inability to work with loss of competence for both employer, and society. The mean fracture-related cost of a distal forearm fracture in Sweden, in patients ≥50 years, during the year following the diagnosis, has been estimated to € 2147 in 2005 [33]. This was however just before the volar locking plate became the dominating surgical treatment [19] and therefore cost per treated patient is supposedly higher now.

As the volar locking plate is a more expensive treatment than other alternatives like casting, wire fixation and external fixation a question needed to put forward is whether it should be the gold standard procedure or not and this has been debated lately. Cost-benefit analyses have revealed small or no gains measured in Quality Adjusted Life Years (QALY) comparing volar plating procedures with other treatment options [34, 35].

With the expected demographic changes with a growing proportion of elderly citizens the number of fractures is likely to increase even if the fracture incidence, despite the findings in this study, should remain stable (Figs. 1 and 2). We have earlier found an increasing incidence also in distal forearm fractures in children [3]. As there are data supporting the idea that childhood fractures are forecasters of future fracture risk [36–38], our projections for the future may underestimate the future number of fractures especially as an incidence increase was also found in working ages in the current study. Interestingly, Mellstrand-Navarro et al. [19] reported that the rate of patients treated operatively with plating increased most (3–4 fold) from 2005 to 2010 in the age group ≥50 years. As our findings suggest an over 50% increase in fractures in the population ≥ 65 years until year 2050 it is of even more interest to make the treatment both evidence-based and as cost-effective as possible (Fig. 2). Therefore further studies are needed.

The strengths of our study include the coverage of a large adult population including all individuals in a well-defined geographical area during 12 years (11.2 million py) with validated data on distal forearm fractures [7]. However, the examination of registers rather than individual patients, charts or X-rays makes selection bias, random or systematic, possible. A previous validation of the register for this particular fracture type has however shown a very high accuracy [7]. Furthermore our results are very similar to those of Brogren et al. from Skåne region [22] and Flinkkilä et al. [16] from Finland who both utilized the gold standard of chart and X-ray review. Even though our case-finding strategy appears valid, estimations of absolute numbers from registers are always difficult. Relative changes (such as time trends) are however more easily subjected to examination and results more robust. We chose a wash-out period of one year, because the vast majority of all distal forearm fractures are then healed and thus, will not appear in the medical records again as a result of that fracture. If an individual appears in the register again, after more than a year with the same diagnosis, the most likely reason is a new fracture and the individual would then be included once more. Bilateral simultaneous fractures will however be counted only as one fracture, as the register does not include information about side. It would have been preferable with in-detail patient level data to identify possible explanatory factors.

## Conclusion

The incidence of distal forearm fractures is increasing in both adult men and women in the Skåne region, Sweden. It is especially worrying that this seems driven by an increase in working ages (17–64 years) as this may present special challenges to both the healthcare system and loss of resources in society during time of sick-leave. The reasons for the increase in incidence are unknown and need further exploration but may include increasing prevalence of osteoporosis and overweight. We suggest similar examinations in other settings with uniform data collection and presentation to verify our results and to enable comparisons and merger of data. Collection and analysis of patient specific risk factors may provide additional insights to the origin of the changes. Cost analysis (including societal costs) and cost-benefit analyses of different treatments in both younger adults and elderly are scarce. Such data would contribute to understanding the effects of our findings and help decision makers to plan best use of resources in the future.

## Abbreviations

BMD: Bone mineral density
BMI: Body mass index
CI: Confidence interval
Py: Person-years
QALY: Quality adjusted life years
SHR: Skåne health care register
